# Characterization and phage-antibiotic synergy of bacteriophages PES-1 and PES-2 against porcine enterotoxigenic *Escherichia coli*

**DOI:** 10.3389/fmicb.2026.1713167

**Published:** 2026-04-22

**Authors:** Tingfang Li, Lina Fu, Jiansong You, Fazal Mehmood Khan, Jianguang Li, Yanfen Liu, Xiaoyu Li, Yongping Xu, Lili Wang

**Affiliations:** 1 MOE Key Laboratory of Bio-Intelligent Manufacturing, School of Bioengineering, Dalian University of Technology, Dalian, China; 2 R&D Center, Aim Honesty Biopharmaceutical Co., Ltd., Dalian, China; 3 College of Science and General Studies, Alfaisal University, Riyadh, Saudi Arabia; 4 Department of Animal Husbandry and Veterinary Medicine, Liaoning Agricultural Vocational and Technical College, Yingkou, China

**Keywords:** antimicrobial resistance, bacteriophage, bacteriophage-antibiotic synergy, enterotoxigenic *Escherichia coli*, food safety, food-borne pathogen

## Abstract

The emergence of drug-resistant porcine enterotoxigenic *Escherichia coli* (ETEC) necessitates the exploration of alternative antibacterial approaches. Two lytic bacteriophages, PES-1 and PES-2, have been isolated and characterized to control ETEC. PES-1 exhibited a wide host range, lysing five ETEC strains, whereas PES-2 displayed restricted specificity, impacting only one of the seven strains. Both phages demonstrated optimal stability at a pH of 4.0–7.0 and a temperature of 4 °C–37 °C. Comparative genomic characterization revealed that the bacteriophages PES-1 have a 40.3 kb size and 48.29% GC contents, while the PES-2 have 67.8 kb size and 46.12% GC Contents, both of which possess a double-stranded linear DNA genomes, lacking tRNA genes, lysogeny-related elements, virulence factors, and antibiotic-resistant factors, emphasizing their obligately lytic lifestyle and genomic biosafety. Genomic analysis revealed that PES-1 exhibited sensitivity to EcoR-I and Hind-III. PES-2 demonstrated resistance to all tested restriction enzymes. Combining bacteriophages with antibiotics such as amoxicillin and neomycin significantly enhanced antibacterial efficacy (*p* < 0.05). A notable synergy was observed with amoxicillin, resulting in a decrease in bacterial loads by 2.02–3.47 log units compared to amoxicillin alone. This synergy likely arises from dual selective pressure and improved antibiotic penetration after phage-mediated lysis. However, no significant difference was observed in the gentamycin-phage combination group compared to the phage cocktail group. The results indicate that PES-1 and PES-2 show environmental stability and effectiveness as biocontrol agents for ETEC. This research introduces an innovative method for developing bacteriophage-based solutions applicable to veterinary and food safety contexts.

## 1 Introduction

Enterotoxigenic *Escherichia coli* (ETEC) is a significant food-borne bacterium responsible for severe diarrhea in humans and young animals, including neonatal piglets, lambs, and calves, representing a significant threat to public health and animal production (Wang et al., 2017; Buuck et al., 2020). ETEC produces enterotoxins, such as the heat-stable (ST) and the heat-labile (LT) enterotoxins, leading to severe watery diarrhea and dehydration, which poses a risk to human health and raises rates of morbidity and mortality, as well as significant financial losses in animal husbandry (Zhou et al., 2015).

Conventional methods depend on antibiotics to prevent and control ETEC. Antibiotics eradicate a wide range of bacterial strains, causing an imbalance in the microbiota of food products, which can result in a marked decrease in beneficial bacteria. This also contributes to the growth of multidrug-resistant (MDR) bacteria, a significant concern for human health and the livestock industry (Khan et al., 2023a). The rise of antibiotic-resistant strains and the growing issue of antibiotic misuse have made the development of new antimicrobial agents an urgent priority (Rello et al., 2016).

As a result, there is a pressing need to develop novel and creative approaches to mitigate foodborne pathogens. Bacteriophages and bacteriophage-derived endolysins present a compelling natural strategy for preventing foodborne pathogens (Lee et al., 2022). In comparison to traditional antibiotics, phage therapy offers several advantages, including a high safety profile, coevolution with bacteria, specificity, ability for self-replication, maintenance of the normal microbiota, bactericidal efficacy, and lower intrinsic toxicity (Bodier-Montagutelli et al., 2017; Domingo-Calap and Delgado-Martinez, 2018; Morrison and Zembower, 2020). Zhou et al. (2015) demonstrated that phage JS09 efficiently lysed ETEC and significantly reduced bacterial proliferation. Cha et al. (2012) combined phages with feed and successfully treated diarrhea in ETEC-infected pigs, with no adverse side effects seen during the experiment. Diarrhea in ETEC-infected pigs was effectively cured by combining phages with feed, and the experiment showed no adverse effects (Jo et al., 2016). While phage therapy has generally been safe and well tolerated in studies to date, a comprehensive understanding of the interactions of phage and human hosts is lacking (Liu et al., 2021).

Recently, the combined use of phages and antibiotics has obtained significant attention due to their highly synergistic effects. This combination strategy can lower the required dosage of antibiotics, slow down the emergence of resistance, and boost antibacterial effectiveness through complementary mechanisms, presenting a hopeful approach for tackling MDR ETEC infections (Gomaa et al., 2025; Yehia et al., 2025).

This study reports the isolation of two lytic phages, PES-1 and PES-2, from animal husbandry and hospital wastewater. These phages specifically target and lyse ETEC. We systematically investigated their morphological characteristics, host range, lytic capacity, tolerance, and synergistic effects with antibiotics. The findings establish a theoretical foundation for developing phages as innovative antimicrobial agents and present new strategies for tackling food safety challenges.

## 2 Materials and methods

### 2.1 Bacteria and sewage

Sewage samples were obtained from a pig farm and a hospital located in Dalian, mainland China. ETEC strains utilized for phage screening were isolated in our laboratory. ETEC-1 and ETEC-2, which harbor genes encoding both heat-labile (LT) and heat-stable (ST) enterotoxins, were chosen as host strains to isolate phages.

### 2.2 Bacteriophage lysate preparation

A 50 mL sewage sample underwent pre-treatment by adding CaCl₂ and MgCl₂ to achieve a final concentration of 1 mmol/L, followed by a 5-min incubation period. Following centrifugation at 8,000 rpm for 10 min to eliminate solids, a 0.22 μm membrane filter was used to filter the supernatant for potential bacteriophage separation. Combine 10 mL of the lysate with 50 mL of log-phase host ETEC bacteria, which have been grown for approximately 5 h to reach a concentration of 10^9^–10^10^ CFU/mL, and incubate overnight at 37 °C. Subsequently, centrifuge the culture at 10,000 rpm for 5 min, collect the supernatant, filter it through a 0.22 μm membrane sterile syringe filter, and store at 4 °C refrigerator.

### 2.3 Bacteriophage identification

For bacteriophage isolation, purification, and propagation, we followed the protocol described by Gomaa et al. (2019) with some modifications. Phage identification was conducted utilizing the double-layer agar technique. The bottom layer consisted of 1.5% agar LB solid medium, which was prepared and stored at 4 °C. The upper layer consisted of 0.5% LB agar medium, which was heated to a molten state and maintained at 55 °C. 10-mL centrifuge tubes were prepared, each containing 1 mL of host ETEC bacterial suspension. The phage suspension underwent serial dilution to a factor of 10^−10^. Subsequently, 10 μL of each diluted phage suspension was introduced into the centrifuge tubes. A control group of 1 mL of host bacterial suspension and 10 μL of LB medium was established. After a 10-min interaction, 5 mL of the upper medium layer was added to each tube, thoroughly mixed, and then poured over the lower layer. The plates were incubated at 37 °C for 18 to 24 h after solidification. The observation of phage plaques indicates the presence of lytic phages that target the host bacteria in the sample.

### 2.4 Bacteriophage plaque purification

Phages underwent purification 3–5 times until consistent plaque morphology and dimensions were attained. Distinct morphologies of individual plaques were selected using sterile 20 μL pipette tips and dissolved in 1 mL of SM buffer. The suspension was gently stirred and incubated overnight at 4 °C to facilitate the release of phages into the buffer. The mixture was centrifuged at 10,000 rpm for 5 min, and the supernatant was filtered using a 0.22 μm membrane syringe filter. The filtrate was serially diluted to 10^−9^ and inoculated into a double-layer agar culture. The procedure was performed 3–4 times until consistently sized and morphologically similar plaques were achieved.

### 2.5 Bacteriophage propagation

Since the purified phage titer was low, liquid culture amplification was necessary. After mixing the bacteriophage lysate with the host ETEC bacteria in a 1:10 ratio, the mixture was incubated for 3–4 h at 37 °C. The amplified phages were collected from the supernatant after it was centrifuged for 5 min at 4 °C at 8,000 rpm.

### 2.6 Phage titer determination

The phage titer was determined using the protocol described by Sada and Tessema (2024) with some modifications. The phage titer, defined as the quantity of phages per unit volume, was calculated using the single-layer agar plate technique. One milliliter of logarithmic-phase ETEC culture was inoculated onto an LB agar plate. The plate was dried after the extraction of the unabsorbed bacterial suspension using a pipette. The phage was serially diluted in PBS, and 10 μL of each dilution was applied to the plate. After incubation at 37 °C for 12–18 h, plaque-forming units (PFUs) were enumerated to determine the phage titer:


Phage titer(PFU/mL)=Number of plaques×Dilution factor×100

### 2.7 Transmission electron microscopy of bacteriophages

Bacteriophages were concentrated using polyethylene glycol (PEG) precipitation (Viazis et al., 2011). The phage was examined under TEM by following the protocol described by Khan et al. (2021) with minor modifications. A concentrated phage suspension was applied to a slide, and a copper grid was placed on top of it. After 10 min, the excess liquid was absorbed using filter paper. A 2% phosphotungstic acid (PTA, pH 7.0) was applied to the grid, allowed to sit for 10 min, and subsequently air-dried. Phage morphology was examined using transmission electron microscopy (TEM).

### 2.8 Optimal multiplicity of infection

Host ETEC bacteria in the logarithmic growth phase were co-cultured with phage at multiplicity of infection (MOI) values of 0.001, 0.01, 0.1, 1, 10, and 100. The mixtures were incubated at 37 °C for 2 h with shaking at 160 rpm. Subsequently, the cultures were filtered through a 0.22 μm filter membrane after being centrifuged at 12,000 × g for 5 min at 4 °C. Phage titers were determined using the double-layer agar plate method. The optimal multiplicity of infection (OMOI) was defined as the MOI yielding the highest phage titer. Burst size was calculated as (final titer − initial titer)/initial titer (Zhang et al., 2026).

### 2.9 One-step growth curve

The One-step growth curve was determined using the protocol described by Manohar et al. (2018) with modifications. Add 200 μL of ETEC to 100 mL of LB broth and incubate for 6 to 7 h until the bacterial concentration reaches approximately 1 × 10^8^ CFU/mL. Introduce phage lysate to 1 mL of the culture at an MOI of 0.1. Incubate at 37 °C for 10 min to facilitate phage adsorption. Perform centrifugation at 6,000 rpm for 10 min at 4 °C. Discard the supernatant and resuspend the pellet in 1 mL of LB broth. Conduct the centrifugation and resuspension procedure two to three times to eliminate unabsorbed phages. Transfer 50 μL of the bacterial suspension into 50 mL of LB broth and thoroughly mix. Prepare a dilution by adding 1 mL of the mixture to 50 mL of LB broth and incubate at 37 °C with shaking at 150 rpm. Determine the phage titer at 10-min intervals utilizing the double-layer plate method. Conduct three replicates for each group and calculate the average of the results.

### 2.10 Host range assay

The single-layer agar plate method was used to assess the phages’ host range according to the protocol described by Gomaa et al. (2019). An LB agar plate was coated with two milliliters of the test bacterial culture. Following drying, 5 μL of each successive dilution of the phage lysate was scattered onto the plate. The phage’s lytic ability against the test bacteria was assessed by observing plaque formation after the plate was incubated for 12 to 18 h at 37 °C.

### 2.11 Phage genome extraction, enzymatic digestion, sequencing, and analysis

The phage genome was extracted as described by Ding et al. (2020). DNase I and RNase A were included in the PEG8000-concentrated phage suspension at final concentrations of 5 μg/mL and 10 μg/mL, respectively, and incubated at 37 °C for 1 h. EDTA (pH 8.0) was incorporated to achieve a final concentration of 20 mmol/mL, followed by adding proteinase K and SDS to final concentrations of 50 μg/mL and 0.5%, respectively. Following incubation at 56 °C for 1 h, the mixture was subjected to extraction using a DNA extraction solution. The nucleic acid extraction solution facilitated a second extraction, after which the genome was precipitated using ethanol and washed with 70% ethanol. The genomic pellet was resuspended in sterile distilled water (Jia et al., 2024). The nucleic acid type was identified by applying restriction enzymes (Xho I, EcoR I, BamH I, HindIII), followed by an analysis of the digestion products utilizing 1.2% agarose gel electrophoresis.

According to Zhang et al. (2026), the isolated genomic DNA of phages PES-1 and PES-2 was subjected to whole-genome sequencing. Open reading frames (ORFs) were predicted and functionally annotated, while tRNA genes were identified and tested for antibiotic resistance and virulence factors. The phage lifestyle was defined, circular genome maps were created, and phylogenetic analysis was performed using the terminase large subunit.

### 2.12 Bacteriophage chloroform sensitivity assay

To assess phage chloroform sensitivity, 0.01 mL of chloroform was incorporated into 1 mL of phage lysate and stirred vigorously according to the protocol described by Sun et al. (2024). The phage titers were listed in Table 1. Following a 10-min incubation, the aqueous phase underwent a tenfold serial dilution. The titer of the aqueous phase was subsequently assessed using the single-layer plate method and compared to the original phage lysate titer to ascertain any alterations.

**Table 1:** Chloroform sensitivity of PES-1 and PES-2.

Phages	Original phage titer (PFU/mL)	Chloroform treatment titer (PFU/mL)
PES-1	3.5 × 10^10^	2.6 × 10^10^
PES-2	11.4 × 10^10^	9.3 × 10^10^

### 2.13 Phage acid–base stability assay

Twelve 50 mL tubes containing 10 mL of phage with the titer of 10^10^ PFU/mL of PES-1 and 10^9^ PFU/mL of PES-2 were prepared for the phage acid–base stability experiment. Each tube’s pH was calibrated to 1.0 to 12.0. The tubes were incubated in a 37 °C water bath for 1 h after comprehensive mixing. The phage titer of each group was subsequently quantified using the single-layer plate method. A graph depicting acid–base tolerance was constructed with pH values on the x-axis and lg (PFU/mL) on the y-axis.

### 2.14 Phage temperature stability assay

In the phage temperature stability assay, seven 50 mL centrifuge tubes, each containing 10 mL of phage with the titer of 10^9^ PFU/mL, were exposed to various temperatures:, 4 °C, 25 °C, 37 °C, 50 °C, 60 °C, 70 °C, and 80 °C for 1 h. After incubation, the tubes were chilled in an ice-water bath. The phage titer in each treatment group was quantified utilizing the single-layer plate technique. The temperature tolerance curve was developed by charting temperature on the x-axis and lg (PFU/mL) on the y-axis.

### 2.15 *In vitro* bacteriolytic activity

During the logarithmic growth phase, the phage was mixed with ETEC-1 bacterial culture at different multiplicities of infection (MOIs: 0.01, 0.1, 1, 10, and 100). All cultures were incubated at 37 °C with shaking at 160 rpm (Sun et al., 2024). A negative control, consisting of an equal volume of PBS buffer in place of phage, was included alongside the infection groups. Additionally, a blank control containing only LB liquid medium supplemented with the same volume of PBS buffer was established. Bacterial concentration in each sample was determined hourly using the colony counting method.

### 2.16 Synergy of phages with other antimicrobials

1. Antibiotic susceptibility assay of ETEC-1

The antimicrobial susceptibility of ETEC-1 isolates was assessed using the K-B method, following the 2024 CLSI guidelines (CLSI, 2024). The tested antimicrobial agents comprised ciprofloxacin, cotrimoxazole, amoxicillin, streptomycin, tetracycline, chloramphenicol, doxycycline, neomycin, polymyxin B, and gentamicin. Based on the antibiotic resistance results, three antibiotics were selected for MIC determination against ETEC-1. Bacterial cultures were adjusted to 0.5 McFarland standard in TTC (Triphenyltetrazolium Chloride) -containing LB medium, then diluted to 100-fold to achieve a final concentration of approximately 5 × 10^5^ CFU/mL. In a 96-well plate, 100 μL of bacterial suspension was mixed with equal volumes of two-fold serial antibiotic dilutions (200, 100, 50, 25, 12.5, 6.25,… μg/mL). After overnight incubation at 37 °C, growth was assessed by TTC color and OD_600_. Where necessary, an intermediate antibiotic concentration between the last red (+) well and the first non-red (−) well was tested in a repeated assay. The MIC was defined as the lowest concentration showing no color change. Controls included LB with water (blank) and ETEC-1 with water (negative) (Veiga et al., 2019).

1. Phage-antibiotic synergy assays

ETEC-1 was grown in LB medium at 37 °C to the logarithmic phase and adjusted to 1 × 10^6^ CFU/mL. One milliliter of the bacterial suspension was mixed with 500 μL of phage cocktail (2 × 10^5^ PFU/mL) and 500 μL of antibiotic solution in sterile glass tubes. The final antibiotic concentrations, based on MIC values, were as follows: amoxicillin 0.2, 0.15, 0.1, and 0.05 μg/mL; neomycin 3.75, 3.5, 3.25, and 3 μg/mL; gentamicin 17.5, 15, 12.5, and 10 μg/mL. After 3 h of incubation at 37 °C, samples were taken, and viable bacterial counts were determined by plate counting. The fold reduction in ETEC-1 CFU was calculated for each treatment group: control, phage cocktail alone, antibiotic alone, and phage–antibiotic combination (Lin et al., 2025).

Treatment groups were prepared as follows: Negative control (1 mL bacterial suspension + 500 μL SM buffer + 500 μL sterile water); Phage cocktail (1 mL bacterial suspension + 500 μL phage cocktail + 500 μL sterile water); Antibiotic: (1 mL bacterial suspension + 500 μL SM buffer + 500 μL antibiotic solution); Phage–antibiotic combination (1 mL bacterial suspension + 500 μL phage cocktail + 500 μL antibiotic solution).

### 2.17 Data analysis

All experiments were performed in triplicate, and the data are expressed as mean ± standard error (SE). Statistical analysis was performed using GraphPad Prism v8.2.1 (GraphPad Software, La Jolla, CA, United States). Statistical significance was assessed using one-way analysis of variance (ANOVA), followed by Tukey’s multiple comparisons test. A *p*-value of less than 0.05 was considered to indicate statistical significance.

## 3 Results

### 3.1 Phage isolation

**Figure 1:**
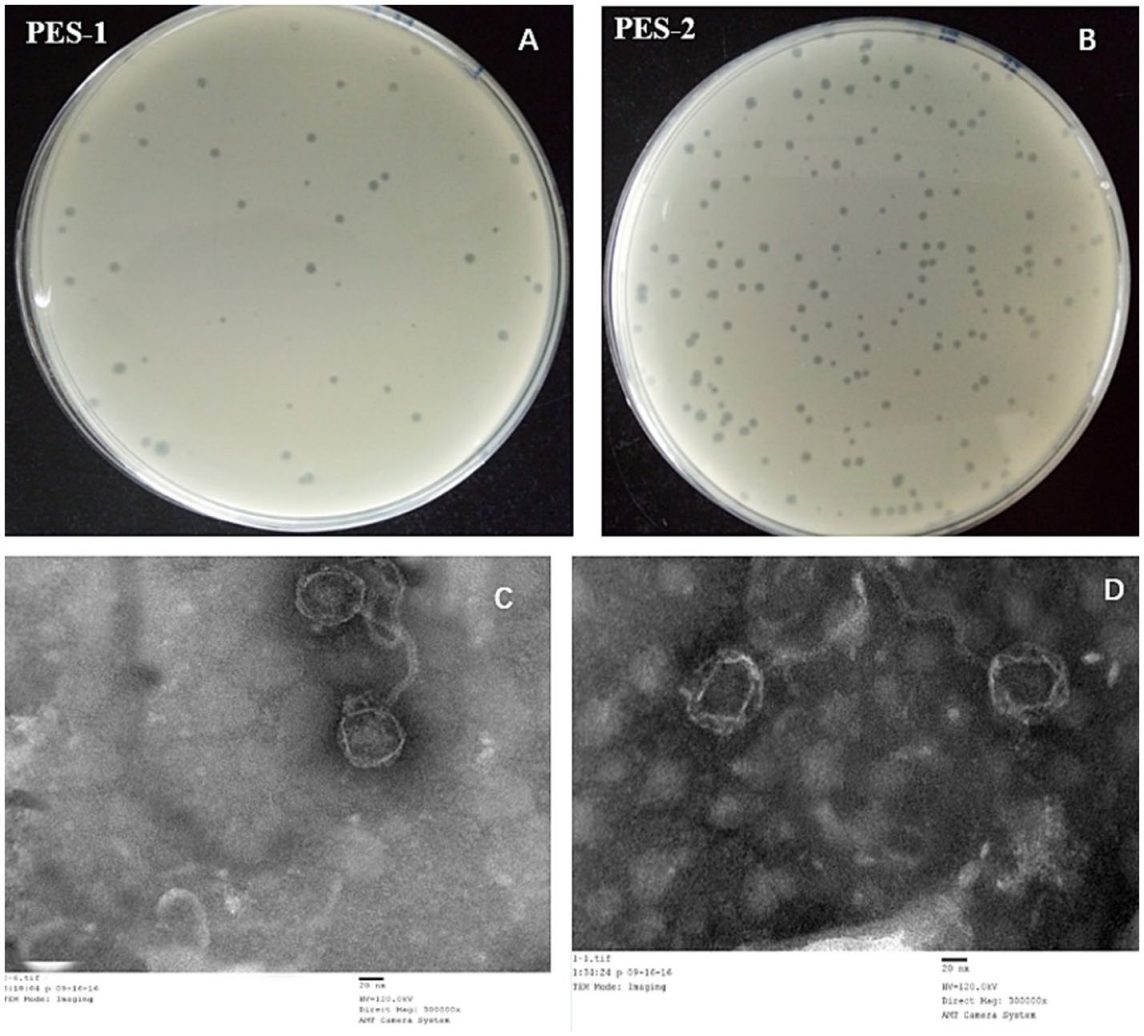
Morphology and electron micrographs of bacteriophage. Phage plaques of PES-1 **(A)** and PES-2 **(B)**; TEM photographs of PES-1 **(C)** and PES-2 **(D)**.

Utilizing ETEC-1 and ETEC-2 as host bacteria, phages PES-1 and PES-2 were obtained from animal husbandry and hospital sewage through the double-layer agar method, respectively, as demonstrated in Figures 1A,B. The phage plaques underwent four rounds of isolation for purification. Ultimately, consistent phage plaques were achieved. PES-1 produces clear plaques with well-defined edges, measuring around 2 ± 0.5 mm in diameter. PES-2 generates smaller, transparent plaques, roughly 1 ± 0.5 mm in diameter.

### 3.2 Transmission electron microscopy of phages

TEM was employed to examine the morphological properties of the isolated phages. The findings indicated that the phages have a head characterized by a regular icosahedral configuration and a non-contractile filamentous tail. PES-1 measures roughly 200 nm in length and 50 nm in width, whereas PES-2 is approximately 150 nm in length and 50 nm in width (Figures 1C,D). The two bacteriophages show no significant morphological differences, additional investigation is necessary to ascertain whether these two phages are classified under the same genus.

### 3.3 Determination of phage host range

Table 2 displays the lytic capacities of phages PES-1 and PES-2 against several bacterial strains. Only one of the seven ETEC strains examined was lysed by PES-2, demonstrating its excellent selectivity and limited host range. PES-1, on the other hand, lysed five ETEC strains, indicating a broader host range and highlighting the need for further study.

**Table 2:** Host ranges of phages PES-1 and PES-2.

Strain	Isolation Source	Strain ID	PES-1	PES-2
ETEC	Pig farm	ETEC-1	+	+
ETEC	Pig farm	ETEC-2	+	−
ETEC	Pig farm	ETEC-3	+	−
ETEC	Pig farm	ETEC-4	+	−
ETEC	Pig farm	ETEC-5	+	−
ETEC	Pig farm	ETEC-6	−	−
ETEC	Pig farm	ETEC-7	−	−
ETEC	Purchased	K88	−	−
*Pseudomonas aeruginosa*	Mink Farm	PA-1	−	−
*Vibrio parahaemolyticus*	Seawater	VB-2	−	−
*Staphylococcus aureus*	Cow Farm	STA-01	−	−
*Streptococcus agalactiae*	Cow Farm	S.a-1	−	−

“+” indicates bacterial sensitivity to the phage; “−” indicates resistance to phage lysis.

### 3.4 Bacteriophage DNA extraction

**Figure 2:**
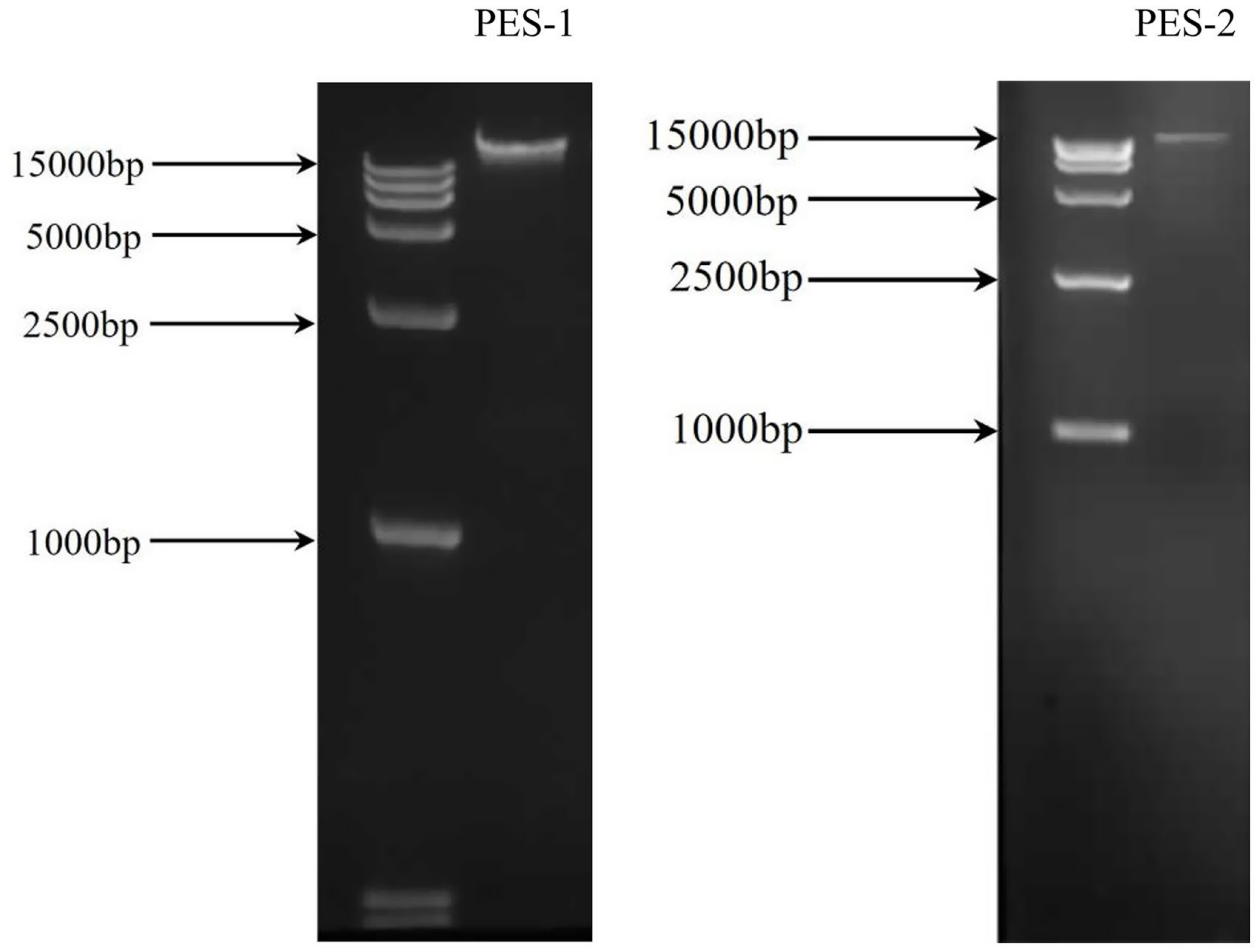
The extracted genomic DNA of phages PES-1 and PES-2.

The genomic DNA of the two phages was extracted and analyzed by electrophoresis on a 1.2% agarose gel. A comparison using a DNA marker indicated that the genomic DNA of both phages surpasses 15 kb (Figure 2).

### 3.5 Bacteriophage nuclease digestion

**Figure 3:**
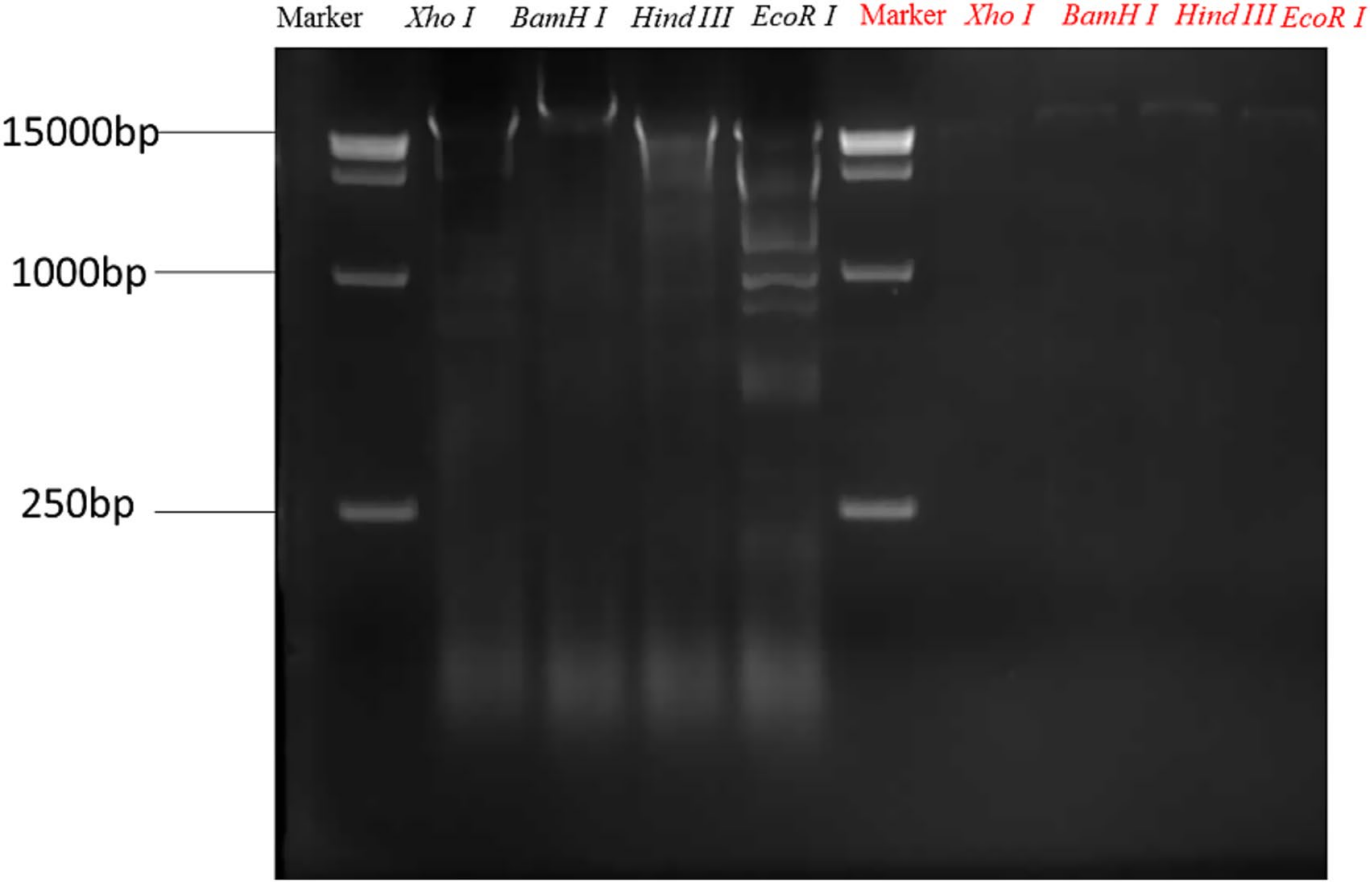
Restriction endonuclease digestion analysis of PES-1 and PES-2 genomes.

Figure 3 demonstrates that of the four restriction enzymes evaluated, PES-1 exhibits sensitivity exclusively to EcoR I and Hind III. Digestion with EcoR I produced five bands, whereas digestion with Hind III resulted in three bands. PES-2 exhibits resistance to all four enzymes. This suggests that the two bacteriophages are genotypically distinct.

### 3.6 Genome analysis

**Figure 4:**
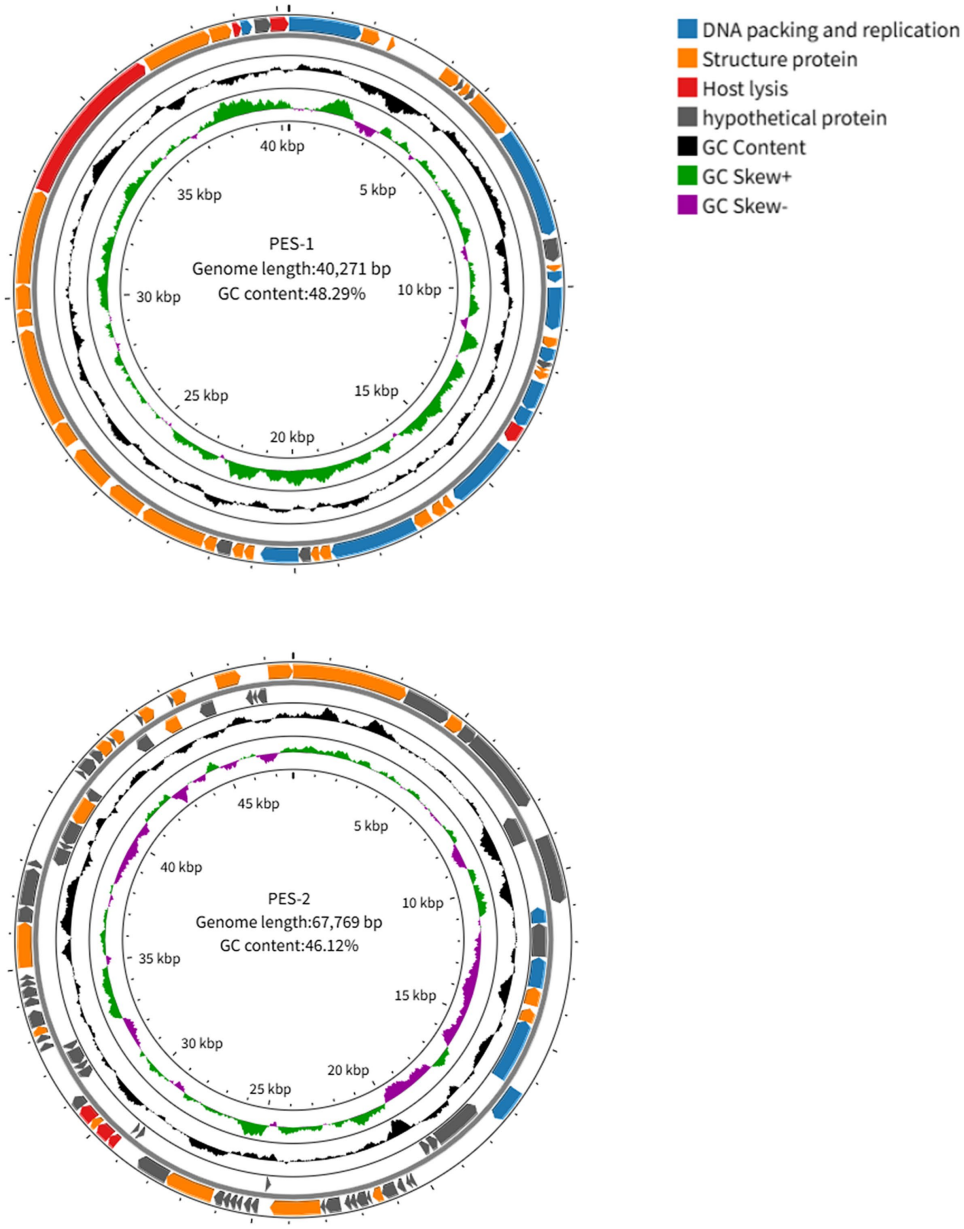
PES-1 and PES-2 annotated genomic maps. An ORF is represented by each colored arrow. The transcription direction is indicated by the arrow’s direction.

The genomes of phages PES-1 and PES-2 are linear double-stranded DNA. PES-1 has a 40,271 bp genome with 48.29 mol% G + C content, whereas PES-2 has a 67,769 bp genome with 46.12 mol% G + C content. The online tRNAscan-SE 2.0 software did not identify any tRNA-encoding genes in the PES-1 and PES-2 genomes. PhaTYP and Bacphlip studies revealed the absence of lysogenic-associated genes in both phage genomes, confirming their classification as virulent phages. Predictions from the online program CARD and VFDB show that the phage genomes lack antibiotic-resistance and virulence genes, implying that the phages are genetically harmless. The findings of the BLASTp and RAST Server prediction models were examined and reported. Phage PES-1 has 49 open reading frames (ORFs), whereas PES-2 contains 84 ORFs. It was discovered that 42 of the 49 ORFs of phage PES-1 have recognized functions. These ORFs are divided into four categories: hypothetical proteins (7 ORFs), proteins linked to phage structure (27 ORFs), proteins involved in phage DNA packaging and replication (11 ORFs), and proteins connected to host lysis (4 ORFs). Of the 84 ORFs in phage PES-2, 25 have known functions. These ORFs were divided into four modules based on bioinformatic features: hypothetical proteins (59 ORFs), proteins involved in phage DNA packaging and replication (4 ORFs), proteins associated with host lysis (3 ORFs), and proteins associated with phage structure (18 ORFs); Figure 4 displays the annotated genome maps of PES-1 and PES-2.

**Figure 5:**
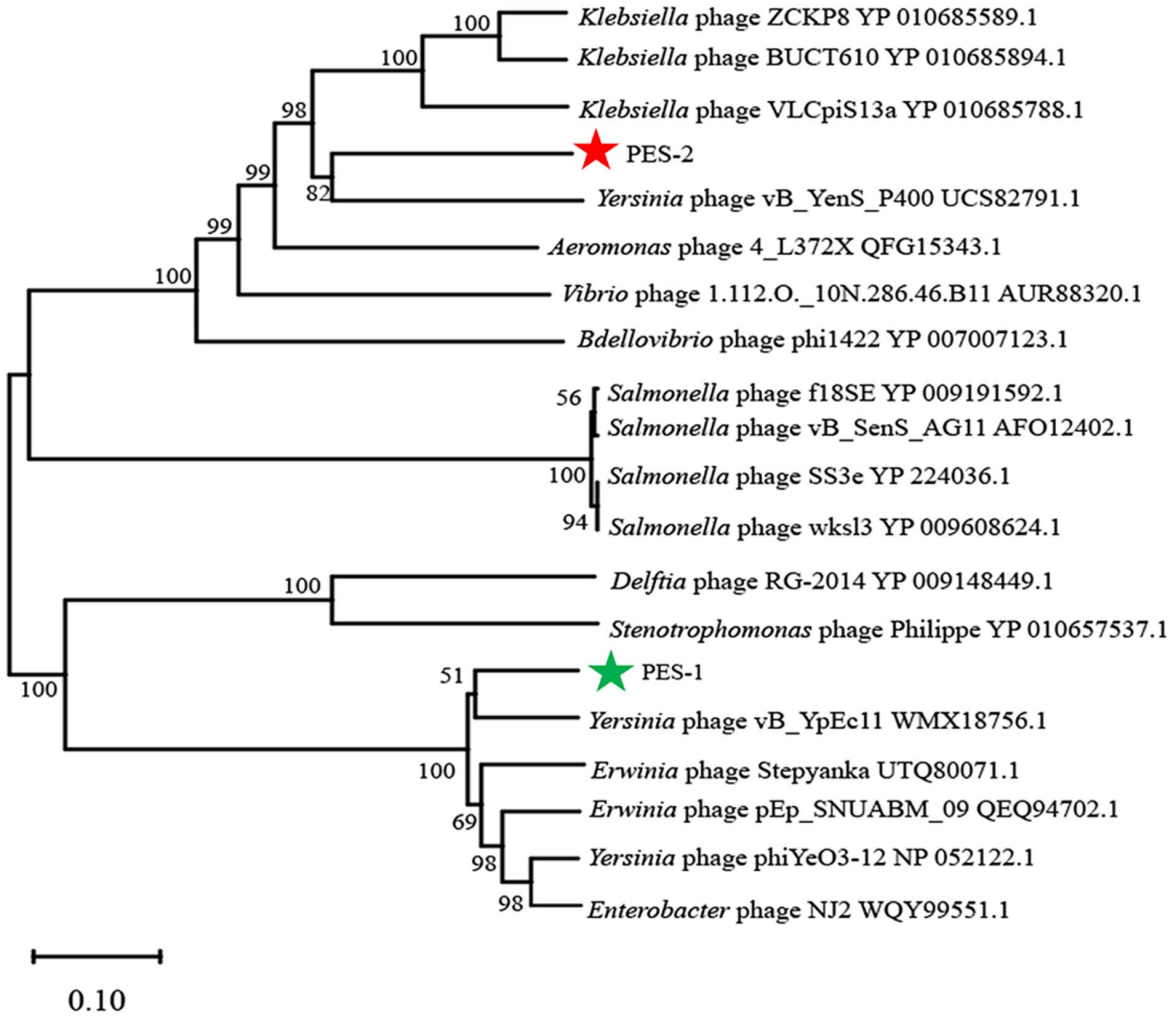
Neighbor-linked phylogenetic trees of PES-1 and PES-2. Alignment with other phages based on the terminase large subunit. The consensus support rate (%) is marked at each node. The green star indicates PES-1, and red star indicates PES-2.

Conserved phage structural proteins, such as terminase’s major component, are widely employed as primary criteria for phage categorization based on expected protein function. The large subunit of terminase is a conserved signature sequence within the phage genome and serves as a primary criterion for phage classification (Turner et al., 2021). To build the phylogenetic tree of phages PES-1 and PES-2, the major subunits of terminase (PES-1: ORF 1, PES-2: ORF 29) were aligned using BlastP. PES-1 has a holin (ORF 46), whereas PES-2 contains lysozymes (ORF 41 and ORF 42). Phylogenetic trees of two phages were generated using the large terminase subunit (Figure 5). Results indicated that phage PES-1 is evolutionarily close to members of the *Straboviridae* subfamily, *Helsettvirus* genus, and PES-2 is closely related to members of the *Peduoviridae* subfamily, *Peduovirus* genus, confirming the classification of the two phages.

### 3.7 *In vitro* characterization of bacteriophage

Based on the host range analysis, ETEC-1—which is susceptible to lysis by both PES-1 and PES-2—was selected as the host strain for subsequent experiments evaluating lytic activity *in vitro* and phage–antibiotic synergy. Table 1 highlights the outcomes of the bacteriophage chloroform sensitivity assay. The titer of PES-1 reduced from 3.5 × 10^10^ to 2.6 × 10^10^ following chloroform treatment, but the titer of PES-2 reduced from 1.14 × 10^10^ to 9.3 × 10^10^. The slight decrease in titer noted for both bacteriophages strongly suggests their significant resistance to chloroform. This resistance indicates that their capsids and tail fibers probably lack lipid components. Moreover, the ability of these phages to withstand chloroform indicates that their structural integrity is maintained in the presence of lipophilic solvents. This chloroform resistance is significant as it highlights their potential applications in the food and agricultural sectors (Gabig et al., 2002).

**Figure 6:**
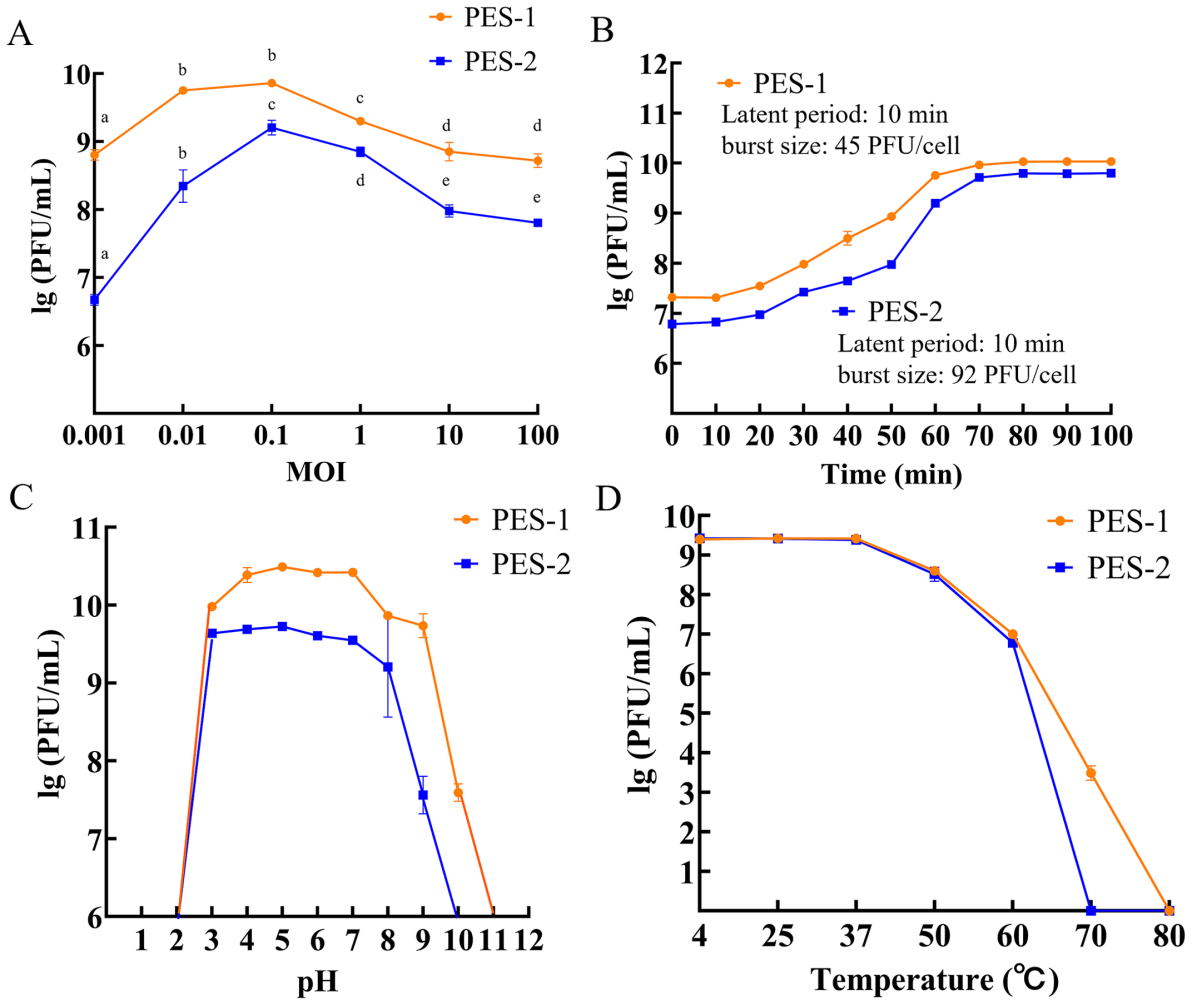
*In vitro* characterization of bacteriophage. Effect of MOI **(A)**, one-step growth curve of phages at OMOI **(B)**, effect of pH **(C)**, and temperature **(D)** on phage viability; different lowercase letters indicate significant differences within the same phage group across different MOIs.

As shown in Figure 6A, the OMOI for PES-1 was 0.01–0.1, while PES-2 achieved maximum titer at an MOI of 0.1. The peak titers of PES-1 and PES-2 were 7.3 × 10^9^ and 1.6 × 10^9^ PFU/mL, respectively. For the one-step growth experiment, an MOI of 0.1 was used for both phages. Results (Figure 6B) showed a latent period of 10 min for each phage, with burst periods of 60 min (PES-1) and 70 min (PES-2). The corresponding burst sizes were 45 and 92 PFU/cell for PES-1 and PES-2, respectively. These properties collectively demonstrate the phages’ capability for fast lysis of ETEC and their efficacy in substantially reducing pathogen populations (Levin and Bull, 2004). Phage stability under different pH conditions is shown in Figure 6C. Both phages remained stable between pH 4.0 and 7.0, but were completely inactivated at pH 1.0 and 2.0. PES-1 was inactivated at pH 11 and 12, while PES-2 already lost activity at pH 10. These results indicate that both bacteriophages are highly sensitive to strongly acidic and alkaline conditions. Thermal stability was assessed across a range of temperatures (Figure 6D). Phage titers remained stable at 4 °C, 25 °C, and 37 °C, decreased at 50 °C, and were completely inactivated at 70 °C (PES-1) and 80 °C (PES-2), demonstrating reduced stability at elevated temperatures.

### 3.8 *In vitro* bacteriolytic activity

**Figure 7:**
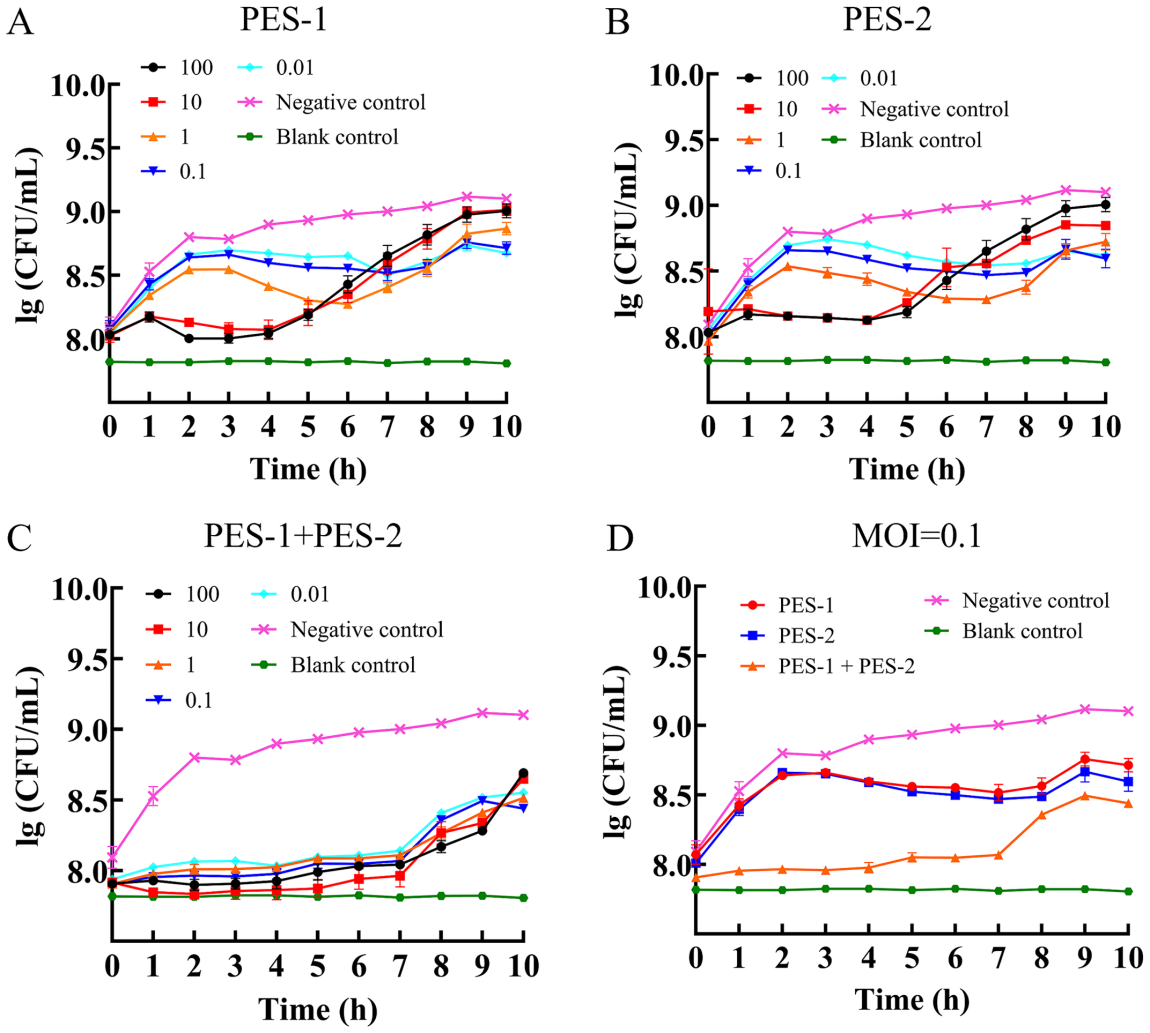
Antibacterial activity *in vitro* of PES-1 **(A)**, PES-2 **(B)**, PES-1 + PES-2 **(C)** at different MOIs, and the antibacterial activity *in vitro* of PES-1, PES-2, PES-1 + PES-2 at OMOI = 0.1 **(D)**.

The *in vitro* lytic activity of PES-1, PES-2, and a phage cocktail against ETEC-1 is shown in Figure 7. At high MOIs (100 and 10), both single phages suppressed bacterial growth for about 4 h, resulting in a decrease in bacterial loads by 0.68–0.80 log units (Figures 7A,B), after which bacterial counts increased sharply until 10 h with a decrease of 0.09–0.25 log units. In contrast, at lower MOIs (1, 0.1, and 0.01), no strong inhibition was observed during the first 3 h (0.09–0.28 log units reduction), but a pronounced antibacterial effect emerged after the fourth hour. By the sixth hour, the MOI = 1 group showed the strongest suppression (0.62–0.66 log units reduction), while from the seventh hour onward, the lower MOI groups (0.1 and 0.01) exhibited more sustained antibacterial activity. At 10 h, the MOI = 0.1 and 0.01 groups demonstrated superior and prolonged inhibition compared to higher MOIs. The phage cocktail inhibited ETEC-1 for approximately 7 h (0.87–1.03 log units reduction) across all MOIs tested (Figure 7C), with bacterial growth resuming thereafter. By the end of the experiment, the strongest overall antibacterial effect was observed at MOI = 0.1 (Figure 7D), which was therefore selected for subsequent phage–antibiotic combination experiments.

### 3.9 Synergistic bactericidal effects of phages with antibiotics

1. Antibiotic susceptibility assay of ETEC-1

The diameters of inhibitory zones were quantified to evaluate ETEC-1’s susceptibility to different antibiotics (Table 3). ETEC-1 was sensitive to cotrimoxazole, amoxicillin, streptomycin, and tetracycline; showed intermediate susceptibility to ciprofloxacin, chloramphenicol, doxycycline, and neomycin; and was resistant to polymyxin B and gentamicin.

**Table 3:** Antibiotic susceptibility profile of ETEC-1.

Antibiotic	Inhibition zone diameter (mm)	Sensitivity
Cotrimoxazole	23	S
Amoxicillin	22	S
Streptomycin	20	S
Tetracycline	18	S
Ciprofloxacin	25	I
Chloramphenicol	14	I
Doxycycline	13	I
Neomycin	9	I
Polymyxin B	0	R
Gentamicin	0	R

“S” indicates susceptible; “I” indicates intermediate; “R” indicates resistant.

Based on the antimicrobial susceptibility profile of ETEC-1, one antibiotic was selected from each category (susceptible, intermediate and resistant), namely amoxicillin, neomycin, and gentamicin, for subsequent combination experiments to assess whether the synergistic effect varies with the degree of bacterial sensitivity. MIC determination revealed values of 0.25 μg/mL for amoxicillin and 4.0 μg/mL for neomycin, while ETEC-1 was resistant to gentamicin. Antibiotic concentrations used in the phage–antibiotic combination assay were then derived from these MIC results.

1. Phage-antibiotic synergy assays

**Figure 8:**
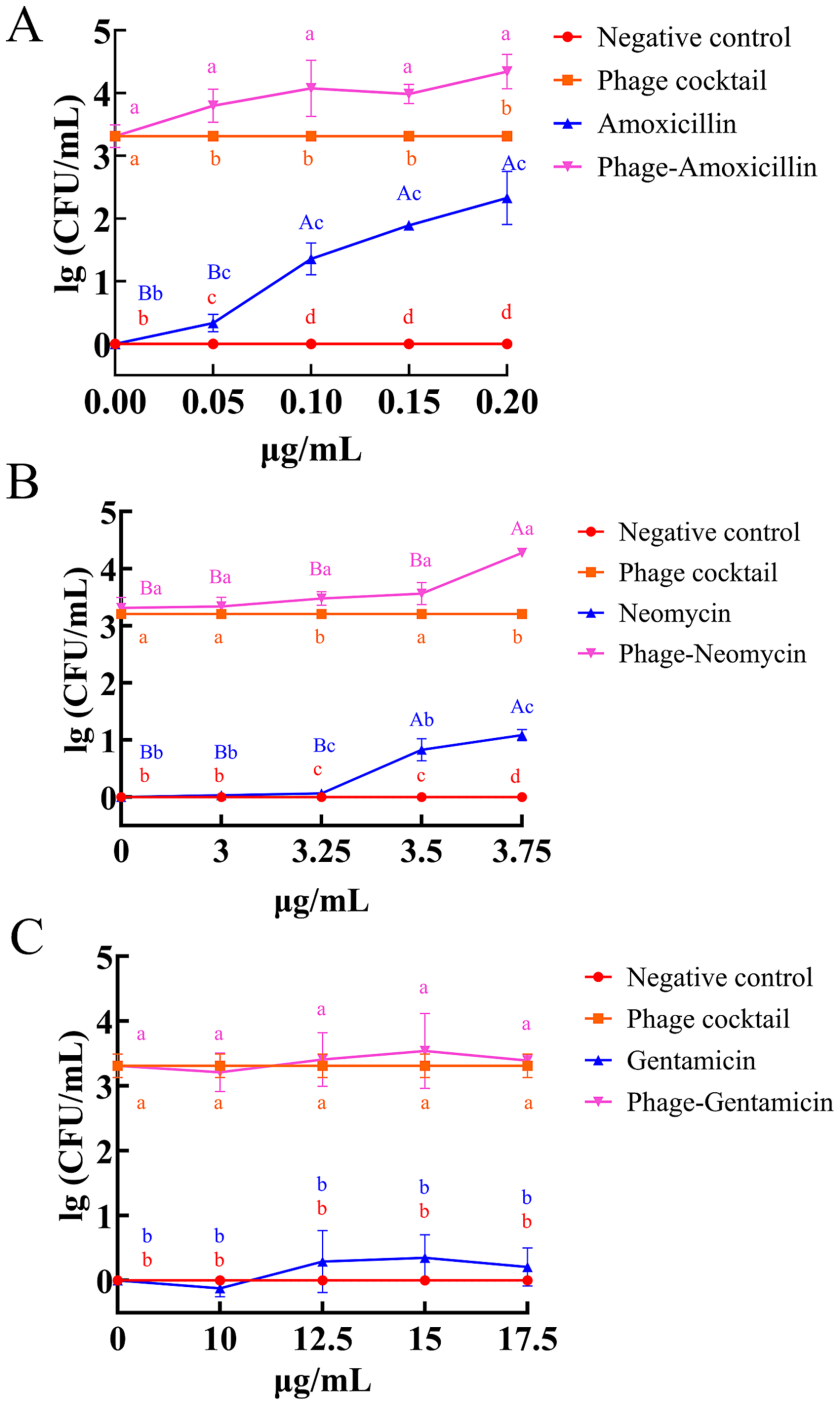
Synergistic bactericidal effects of phages with antibiotics. Fold reduction of ETEC-1 CFU by the combined application of phage cocktail and amoxicillin **(A)**, neomycin **(B)**, and gentamicin **(C)**. Data are expressed as mean ± standard deviation (*n* = 3). Means within a column that do not share the same letter differ substantially (*p* < 0.05). Different lowercase letters indicate significant differences between groups at the same antibiotic concentration, while different uppercase letters denote significant differences within the same group across different antibiotic concentrations.

Figure 8A shows the results of the amoxicillin-phage combination assay. Amoxicillin alone inhibited ETEC-1 in a dose-dependent manner, with reductions ranging from 0.33 log at 0.05 μg/mL to 2.33 log at 0.20 μg/mL (*p* < 0.05). The phage cocktail alone reduced bacterial concentration by 3.31 log, exhibiting stronger antibacterial activity than any single amoxicillin concentration. The combination of amoxicillin and the phage cocktail yielded greater reductions in ETEC-1 levels than either treatment administered alone. Compared to the phage-only group, the combination reduced bacterial counts by an additional 0.49–1.03 log across the amoxicillin concentrations tested. At all concentrations, the combination treatment showed significantly greater antibacterial efficacy than either monotherapy (p < 0.05), indicating a synergistic interaction. The strongest enhancement was observed at 0.20 μg/mL amoxicillin.

Figure 8B shows the results of the neomycin–phage combination assay. Neomycin also exhibited dose-dependent inhibition, reducing ETEC by 0.83 log at 3.5 μg/mL and 1.09 log at 3.75 μg/mL, with no significant difference between these two doses. Neomycin at 3.25 μg/mL or lower showed no detectable antibacterial effect against ETEC-1. The phage cocktail, however, exerted significantly stronger antibacterial activity (3.31 log reduction) than neomycin alone (*p* < 0.05). Even at the highest neomycin concentration tested (3.75 μg/mL), the phage cocktail still achieved a 2.22 log greater reduction in bacterial load compared with neomycin alone. The combination of phage and neomycin further enhanced antibacterial efficacy relative to the phage-only group, with significant improvements observed at neomycin concentrations of 3.25 μg/mL and 3.75 μg/mL (*p* < 0.05). The maximum reduction in ETEC-1 concentration reached 4.28 log units, achieved with the combination at the highest neomycin concentration of 3.75 μg/mL.

ETEC-1 demonstrated resistance to gentamicin, showing only a weak antibacterial effect even at high concentrations between 12.5 and 17.5 μg/mL, with no significant difference compared to the negative control group (Figure 8C). Moreover, when the phage cocktail was combined with gentamicin at high concentrations, a modest enhancement in antibacterial activity was observed. However, this improvement was not statistically significant relative to the treatment with the phage cocktail alone.

## 4 Discussion

Antibiotics have been the main way to prevent and treat bacterial infections, including those caused by Enterotoxigenic *Escherichia coli* (ETEC). However, the inappropriate use and excessive application of antibiotics in both human medical and livestock fields, especially in cattle, poultry, and pigs, have caused the rapid spread of drug-resistant bacteria (Marshall and Levy, 2011; Loh et al., 2021). The WHO has emphasized the pressing need to develop new antibacterial medicines to address the significant gap caused by the emergence of high rates of resistance and an underdeveloped antibiotic development pipeline (Abdelkader et al., 2022).

This pressing situation has intensified the search for alternative antibacterial strategies. Bacteriophages have emerged as a promising natural and specific antimicrobial option (Khan et al., 2023b). Their ability to target specific bacteria without disrupting beneficial microbiota, combined with the potential for synergy when used alongside antibiotics, offers a novel approach to overcoming multidrug-resistant infections (Loh et al., 2021).

In this study, the isolation and characterization of bacteriophages PES-1 and PES-2 contribute to the development of alternative strategies against porcine enterotoxigenic *Escherichia coli* (ETEC), especially in combating antibiotic resistance. This research demonstrates that these phages exhibit strong lytic activity, environmental resilience, and potential synergism with conventional antibiotics, making them a viable approach for managing ETEC in swine production.

The two phages differ significantly in genome size (40,271 bp vs. 67,769 bp), G + C content, and functional annotation rates, with PES-2 containing a notably high proportion (70.2%) of hypothetical proteins. Phylogenetic analysis based on the large terminase subunit places PES-1 within the subfamily *Straboviridae*, genus *Helsettvirus,* and PES-2 within the subfamily *Peduoviridae*, genus *Peduovirus*. Although PES-1 encodes a holin and PES-2 encodes lysozymes, neither phage alone possesses a complete holin-lysozyme system; nonetheless, both demonstrated potent lytic activity against ETEC in subsequent experiments, indicating the involvement of alternative lysis pathways or the activation of host autolysins (Sun et al., 2025). Given their different lytic components, the simultaneous use of the two phages may provide synergistic effects. Both phages lack tRNA genes, lysogenic-associated genes, antibiotic resistance genes, and virulence factors, which supports their classification as virulent phages and confirms their genetic safety for prospective therapeutic uses.

The unique host range characteristics of PES-1 and PES-2 suggest various potential applications. PES-1 demonstrated broad-spectrum efficacy against five of the seven ETEC strains, while PES-2 exhibited notable specificity for one particular strain. PES-1 has potential as a therapeutic agent; however, PES-2’s specificity may enable targeted therapy while minimizing effects on commensal microbiota (Cong et al., 2021). The phages demonstrated suitability for various agricultural and veterinary applications due to their sustained efficacy under physiological conditions (pH 7.0, 37 °C) and their functionality within a therapeutically relevant range (pH 4.0–7.0, 4 °C 37 °C) (Zhang et al., 2026). Chloroform resistance indicates structural integrity, likely due to a lipid-free capsid composition that improves stability under various environmental conditions (Sun et al., 2024).

Phages with a latent time of 10 min and a burst size of approximately 45 and 92 PFU/cell for PES-1 and PES-2, respectively, which demonstrate high lytic efficiency, suggesting beneficial practical applications (Comeau et al., 2008). The optimal antibacterial activity was observed at MOIs of 0.1 and 0.01 for PES-1, and at an MOI of 0.1 for PES-2. The rapid replication cycle suggests that low infection multiplicities may be advantageous, as they lower production costs and enable widespread applications in the food industry and agriculture (Khan et al., 2024; Sun et al., 2025).

The study demonstrated that these phages synergize with amoxicillin and neomycin. Phage-antibiotic combinations reduced bacterial loads by 2.02 to 3.47 log units compared to antibiotics alone. Gomaa et al. (2025) also found that phage Henu12 acts synergistically with polymyxin B, tetracycline, and ceftazidime against *Shigella dysenteriae*. Multiple pathways contribute to the synergistic effect: First, the combination of antibiotic and phage lowers the metabolic activity of bacteria. By interfering with cellular metabolic processes, impairing bacterial growth and repair capacities, and increasing phage bactericidal efficacy, the combination inhibits bacterial metabolism more than phages alone (Qin et al., 2024). Additionally, a synergistic bactericidal effect is observed. In conjunction with lytic proteins such as CHAPSH3b, phages degrade extracellular polysaccharides (EPS), exposing underlying bacteria and enhancing antibiotic penetration. Vancomycin targets peptidoglycan within the cell wall. Antibiotics modify bacterial physiological characteristics, including cell morphology, which increases phage adsorption (Coyne et al., 2024). Third, dual selective pressure necessitates that bacteria develop resistance to both phage infection and the effects of antibiotics concurrently. This elevates the evolutionary cost and reduces the frequency of drug-resistant mutations, thereby effectively inhibiting the development of drug resistance in pathogens (dos Reis et al., 2021).

Interestingly, amoxicillin exhibits inhibitory activity against both Gram-positive and Gram-negative bacteria; however, the degree of susceptibility varies among bacterial species. This variability is primarily attributed to differences in cell wall architecture, the number and types of penicillin-binding proteins (PBPs), and the permeability characteristics of the outer membrane (Jacoby and Munoz-Price, 2005). In *Escherichia coli*, amoxicillin demonstrates strong antibacterial activity due to the presence of outer membrane porins such as OmpF and OmpC, which facilitate its diffusion across the outer membrane (Delcour, 2009). Amoxicillin binds to PBPs in *E. coli*, inhibiting cell wall synthesis (Sauvage et al., 2008). This inhibition can stimulate endogenous autolytic enzymes, leading to cell wall degradation and bacterial death (Tomasz, 1979). Phage-induced membrane disruption enhances antibiotic penetration, while concurrent selective pressures from both agents increase the evolutionary barrier to the development of resistance (Comeau et al., 2008; Yehia et al., 2025). Furthermore, metabolic disruption caused by antibiotics increases bacterial susceptibility to phage predation (Gurney et al., 2020).

The findings of this study extend and complement prior research on phage therapy for ETEC control, and the genomic characteristics of PES-1 and PES-2, particularly their unique restriction enzyme sensitivity profiles, suggest their potential for application in controlling porcine *Escherichia coli* infections. Phages can function as feed additives, prophylactic agents, or interventions in meat processing, thereby improving veterinary health and food safety. The restricted host range and temperature sensitivity of PES-2 at temperatures exceeding 70 °C may require the implementation of phage cocktails or tailored formulations for specific applications.

Future research should focus on comprehensive genome sequencing to assess virulence and lysogeny traits, validate *in vivo* efficacy in animal models, and enhance delivery methods, particularly through the use of encapsulation technology. The interaction between phages and antibiotics, especially in restoring effectiveness against resistant bacteria, makes antimicrobial management in porcine production essential. Integrating phage-based approaches with existing management techniques can improve the sustainability and effectiveness of ETEC control strategies, influencing animal, food, and public health. This study suggests that phage therapy may be a viable alternative to antibiotics in agricultural and veterinary settings.

## Data Availability

The raw data supporting the conclusions of this article will be made available by the authors, without undue reservation.
